# Service of Prof. E. Andrews, M.D., Professor of Surgery in the Medical Depart. Lind University

**Published:** 1860-12

**Authors:** 


**Service of Prof. E. ANDREWS, M.D., Professor of Surgery in the Med. Depart. Lind University.**

### *New Instruments for the Treatment of Hip Disease.—Remarks and Cases Illustrating their Use.*

GENTLEMEN:—

The treatment of *morbus coxarius* has undergone a great revolution within the last eighteen months. Before that time, it is true that surgeons understood the pathology of the disease, and employed, to ameliorate it, remedies which were at once rational and useful; but the difficulties, of a practical nature, which beset treatment, were such that in general the remedial measures were shorn of their efficiency, and almost reduced to the low grade of palliatives. The ends to be aimed at in this disease are: 1st, To correct the constitutional diathesis; and 2nd, To remove the local causes of irritation. In the constitutional treatment no material improvement has, of late, been made; but in the local measures, revolutionizing changes have been effected. The obstacles which have hitherto stood in our way in managing this disease, were: 1st, The excessive dread of any operative procedure which should open the cavity of the joint; and, 2nd, The difficulty of preserving the inflamed synovial surfaces from friction against each other, without confining the patient to the bed for such a length of time as must prove disastrous to the constitutional vigor.

Those who have read the current surgical literature of the country for a year past, will readily recall the following conclusions, which are now admitted by the best surgeons:

1st. In the primary stages of the disease there is often an excess of synovia secreted, which, by its presence in the confined sac of the capsular ligament, greatly increases the pain and exasperates the inflammation. It is now deemed advisable to tap the joint and evacuate the fluid in such cases. This is usually accomplished by introducing a small trochar through the capsular ligament behind the great trochanter. If there is an accumulation of pus in the cavity, it is evacuated in the same way. In this manner the parts are relieved, and in many instances the destruction of bone is prevented. 2d. If, however, the bone has actually become carious, it is deemed best to operate at once, and remove the diseased portion by excision of the head of the bone. It is found that patients bear this operation well, and that the risk is a mere trifle compared with the appalling danger of leaving the sequestrum for years as an irritant and provoker of exhausting suppuration. The old objection that the ilium *may be* involved in the caries, has no force. Usually that bone is sound, and when otherwise it is often affected only around the acetabulum where the gouge readily removes the diseased part. In short, we remove carious and necrosed bone from this region, with just as little hesitation as from any other part of the body. The tapping of the joint in some cases, and the excision of the diseased bone in others, are finally established as important remedies in this disease.

In addition to these improvements, we have equally important ones in the mechanical appliances made use of.

Formerly the long splint, or the starch bandage, combined with rigid confinement to the bed, were the only measures by which we were able to prevent the friction and pressure of the inflamed surfaces against each other. The objections to these measures are: 1st, That the confinement to the bed is disastrous to a constitution already half ruined by debility. 2nd, That so far as the starch bandage is concerned, it only relieves the *friction* of the joint, but does not diminish the *pressure* on the diseased surfaces. To accomplish these indications better, Drs. Davis and Sayres, of NewYork, have each produced a splint, one being a modification of the other. I have been able to see only one of these which I will new describe. It consists of a steel splint fitted to the curves of the outer side of the hip, thigh and leg. It is attached to the leg by adhesive straps, and to the hip by a perineal band It is provided with a ratchet, &c., for the purposes of extension. With this apparatus the limb is kept extended so that the pressure of the inflamed head of the femur into the acetabulum is completely taken off. The patient is not confined to his bed, but allowed to go about, taking air and exercise, and invigorating in this way his general health.

In making use of a similar apparatus, however, I find the following inconveniences:

1st. The splint, in order to make proper counter-extension, necessarily reached high above the hip joint. It therefore operates to prevent the flexing of the thigh on the body to such an extent as to render all motions of sitting down and rising up very difficult.

2nd. When applied after excision of the head of the bone it comes over the seat of the wound in such a way as to not only become fouled by the discharges, but to very much interfere with the cleansing and dressing of the limb.

On this account I have sought to modify the principle, and have devised the instrument, which you here see. It consists, you see, of a rod of iron, to be applied to the *inner side* of the leg and thigh, with a foot piece, which is riveted to the sole of the shoe. To the upper end is attached a sliding rod and screw for extension, surmounted by a crutch top well padded, which rests against the perineum. In adjusting it the foot is placed in the shoe and held there by long and broad adhesive straps attached to either side of the leg and brought down and tied or sewed under the foot piece. The screw is then turned up until the padded crutch top rests firmly against the perineum, and the desired extension is accomplished. In this way the weight of the patient rests upon the instrument and the instrument upon the ground, without impairing the extension. The superior extremity not reaching above the joint, the patient readily flexes the thigh when sitting, and the instrument being on the inner side of the limb, is out of the way of any foul discharges after the operation of excision.

Case 1.—This little girl was presented to you some months ago in the very first stages of the disease. She then discontinued her attendance for a time, but again gradually getting worse, she returned. The left lower extremity was as usual drawn up in the vain instinctive effort to lift the acetabulum away from the torturing pressure of the head of the femur. There was the usual pain in the knee and a general excitement and irritation of the nervous system; but no evidence of actual caries. I applied at once this instrument, keeping it on night and day. From that hour the patient began to improve. The joint, relieved from pressure, grew less inflamed, the pain subsided, and the apparent shortening disappeared. At the present the patient walks about upon the instrument without suffering, and is in a fair way to recover with a perfect limb.

Case 2nd.—This man lay in the wards a long time, with obscure symptoms resembling rheumatism. At length, however, there were evidences of a suppurative diathesis; the disease manifested its location in the hip joint. The limb took on all the usual appearances of hip disease in the early stages, and I considered the resemblance sufficient to call for the same treatment. Accordingly the instrument was constructed and applied. The result was most gratifying. The soreness from that time began to subside, the limb came down to its place, and now the patient walks freely on his instrument without a particle of suffering. You will observe by pressure upon the dorsum of the ilium, a hard projecting ridge, showing that there has been extensive periostitis around the joint, and that had it not been for this mode of treatment he would doubtless be now laboring under the evil of a carious hip joint.

Case 3rd.—This little girl was first brought under my care with the bone already necrosed. She was thin, pale, exhausted by suppuration and tortured by pain. I excised the superior extremity of the femur. The ilium was found healthy and required no gouging; but the head of the femur was entirely gone, and several sequestra ledged in the shaft. After the operation the apparatus was applied as in the former cases. The pain very soon disappeared entirely; the patient gained flesh and strength, and now walks with comfort upon the instrument. By continuing the use of it we shall prevent the limb from being much shortened, and after the femur has contracted a tolerably strong ligamentous attachment to the ilium, the artificial support may be left off, and we shall see that there will be a surprisingly good use of the limb in walking notwithstanding the loss of substance in the bone. I have other cases on my hands, but they are not yet sufficiently advanced to enable me to report their results to you.

The instrument costs only about three dollars for the iron work, if made in a plain way. The padding of the crutch piece, which can be done by any ingenious seampstress, should be covered with oiled silk, or sheet india-rubber, to prevent the absorption of perspiration or other secretions.

Thus, gentlemen, you will find in your experience that *morbus coxarius* is a disease which has lost a great part of its terrors. By the measures which I have explained to you, it will yield to your skill, and you will find yourselves able hereafter to save many a life such as your predecessors have been wont to lose.

